# National physical activity recommendations: systematic overview and analysis of the situation in European countries

**DOI:** 10.1186/s12889-015-1412-3

**Published:** 2015-02-12

**Authors:** Sonja Kahlmeier, Trudy M A Wijnhoven, Patrick Alpiger, Christian Schweizer, João Breda, Brian W Martin

**Affiliations:** Physical Activity and Health Unit, Epidemiology, Biostatistics, and Prevention Institute (EBPI), University of Zurich, Seilergraben 49, 8001 Zurich, Switzerland; Nutrition, Physical Activity and Obesity, Division of Noncommunicable Diseases and Promoting Health through the Life-Course, World Health Organization (WHO) Regional Office for Europe, UN City, Marmorvej 51, DK-2100 Copenhagen Ø, Denmark; Environment and Health, Division of Communicable Diseases, Health Security and Environment, WHO Regional Office for Europe, UN City, Marmorvej 51, DK-2100 Copenhagen Ø, Denmark

**Keywords:** Physical activity, Public health, Recommendations, Guidelines, National policy, Review, Comparative analysis, Europe

## Abstract

**Background:**

Developing national physical activity (PA) recommendations is an essential element of an effective national approach to promote PA.

**Methods:**

Systematic overview and analysis of national PA recommendations across the European Region of the World Health Organization (WHO). The WHO European national information focal points provided information which was complemented through online searches and input from other experts.

**Results:**

Information received until summer 2012 from 37 countries was analyzed. Sixteen countries did not have national recommendations while 21 countries did. For 17 countries, the source document was accessible. Seventeen recommendations referred to adults, 14 to young people and 6 to older adults. Most national recommendations for children and young people are quite similar: 12 countries recommend at least 60 minutes of moderate- to vigorous-intensity PA each day, in line with the WHO global recommendation. Three countries recommend longer durations and one a lower one. In some countries, slight variations were found regarding the recommended intensity and minimum bouts. Only one country was fully in line with the WHO recommendations. Two countries have issued separate recommendations for pre-school children. For adults, most countries still follow the 1995 United States recommendations of “at least 30 minutes on 5 days a week”. Three countries were fully in line with the WHO recommendations. Four countries give specific recommendations on reducing weight, avoiding weight gain or continuing weight maintenance. The six identified national PA recommendations for older adults are mainly similar to those for adults but underline that particularly for this age group also less activity has important health benefits; four countries also recommend balance training.

**Conclusions:**

About half of the countries for which information was available and likely less than 40% of all 53 countries in the WHO European Region have developed national PA recommendations. Further investment is needed to address this important step towards a comprehensive PA promotion approach. Much remains to be done for the 2010 WHO recommendations to be fully reflected in national documents across all parts of the Region and all age groups. In addition, avoiding extended periods of inactivity and overweight are only addressed by a minority of countries yet.

## Background

Since the landmark report of the United States Surgeon-General on physical activity (PA) and health 17 years ago [1] and the adoption of the Global Strategy on Diet, Physical Activity and Health by the World Health Assembly in 2004 [2], PA has increasingly been recognized as a key determinant for good health across Europe as well as globally. Insufficient levels of PA are prevalent, with almost two thirds of adults and 80% of young people not reaching the minimum recommended levels of PA in Europe [3]. Lack of PA has become the fourth-leading risk factor in western Europe and other high-income regions and is among the top 10 globally, being associated with about 3 million deaths per year [4] as well as 6–10% of the major noncommunicable diseases (NCDs) such as coronary heart disease, type 2 diabetes, and breast and colon cancers [5]. Recent international policy frameworks acknowledged the importance of PA [6-9]. Furthermore the Global Monitoring Framework for NCD [10] established a global target of 10% reduction in physical inactivity by 2025.

While its importance is now well recognized, PA promotion is still a rather young field, compared to other risk factors for NCDs, such as diet or tobacco use [11]. Nevertheless, in recent years more evidence has emerged on characteristics of effective policies for PA promotion. WHO has published policy guidelines to support countries in developing comprehensive and effective national policies [12,13] as well as the European Commission (EC) [14]. Bellew et al. proposed the so called “HARDWIRED” policy criteria [15] and actions to promote PA [16] and recently, a health-enhancing physical activity (HEPA) policy audit tool was presented [17,18], structured around a set of 17 key attributes identified as essential for successful implementation of a population-wide approach to PA promotion across the life course. One element proposed in all of these guidelines is the development of national PA recommendations. Their development can bring together all relevant stakeholders and actors, their launch helps to bring PA onto the public agenda and they serve as benchmark for the implementation of a national policy and related programmes and projects.

Much progress has been made on the evidence of the amount, frequency and intensity of PA to achieve comprehensive health effects since the publication of the first PA recommendations by the United States Centers for Disease Control and Prevention (CDC) and the American College of Sports Medicine (ACSM) in 1995 [19], which have had an impact in many other countries around the world. An extensive review of new evidence on the health effects was undertaken [20], and updated recommendations were issued by the United States Department of Health in 2008 [21] and by WHO in 2010 with the overall aim of providing policy makers with guidance on the dose–response relationship between the frequency, duration, intensity, type and total amount of physical activity needed for the prevention of NCDs [22]. It has been suggested that a review of the current recommendations in Europe should be undertaken in view of the latest international recommendations [23]. This paper provides an overview of the state of affairs on national PA recommendations across the WHO European Region, and an analysis of their content compared with the international recommendations.

## Methods

A combination of different approaches was used to collect information on national PA recommendations. As part of a joint WHO/EC project in 2008–2011, a country reporting template was prepared to gather information on a range of topics, including on policy documents aimed at counteracting obesity, and those focusing on the promotion of healthy nutrition or PA as well as on national recommendations [24,25]. This template was sent for completion to all 53 Member States in the WHO European Region in 2009/2010; the WHO national information focal points from 44 countries replied. When the provided information was incomplete, outdated by summer 2011 (e.g. links provided were no longer functional or a reported document was not retrievable) and could not be updated through an online search for the document, the official WHO country focal point or relevant national experts were contacted for additional information in autumn 2011 and further information was verified until summer 2012. In addition, confirmation was sought for those countries where the focal point had replied that national PA recommendations had not yet been developed.

In order to analyze official national recommendations, documents were included if an officially adopted written statement on the frequency, duration and intensity of PA needed to achieve health benefits existed and the source document was retrievable. Recommendations were not included if they had been issued by a nongovernmental organization or a national institute of public health but were not officially endorsed by a government body, or if they were only a general suggestion to be physically active. Documents were included if they existed in English, German and French.

An analysis grid was developed for standardized analysis and comparison of the national recommendations against one another as well as against the 2010 WHO recommendations. Since this review was finalized only two years after WHO [22] and four years after the United States Department of Health [21] released its recommendations, also the 1995 CDC/ACSM recommendations were used for comparison [19]. Besides the basic elements of the minimum recommendations (frequency, duration, intensity), analysis also included:

- Any statement on minimum duration of bouts of PA;
- Further recommendations, e.g. for additional health benefits, strength, balance and bone health training or overweight prevention;
- Specific recommendations on extended periods of inactivity or sitting.

The recommendations were analyzed by age groups (children/young people, adults, older adults/elderly), as available.

## Results

**Figure 1:**
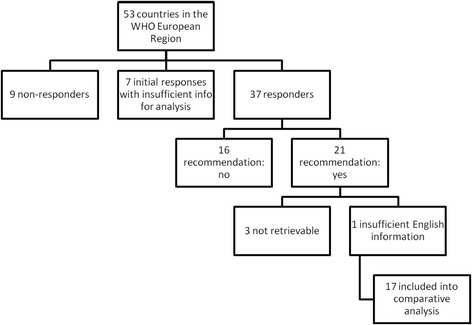
**Flow diagram of identified and included national recommendations from the WHO European region.**

Information about national PA recommendations was found for 37 of the 53 countries in the WHO European Region (69.8%) (see Figure 1). Of these, 21 countries reported to have developed national PA recommendations including a statement on the frequency, duration and intensity of PA needed to achieve health benefits (56.8% of countries for which information was available and 39.6% of all 53 countries in the European Region). For 18 countries, the source document was accessible; documents reported for Hungary, Lithuania and Romania were not retrievable. In addition to recommendations in French and German (France, Switzerland) amenable to the study team, for four countries, the national PA recommendations were only available in the national language. In the cases of Iceland, the Netherlands and the Russian Federation, the national WHO focal point or national experts provided additional information for the analysis grid, which could be verified by translating key terms with the Google online translation tool. These three countries were thus also included into the analysis while for the identified Estonian document insufficient information was available for detailed analysis. Thus, for 17 countries the content could be analyzed in detail and compared with the international recommendations (see Table 1). The national recommendations of Norway and Sweden are similar as they refer to the same source document [26], which was developed by the Nordic Council of Ministers that both countries are members of. From 16 responding countries, confirmation was received that national PA recommendations have not been developed yet. These countries were Albania, Andorra, Azerbaijan, Bosnia and Herzegovina, Croatia, Georgia, Germany, Italy, Latvia, Poland, Portugal, Serbia, Spain, the former Yugoslav Republic of Macedonia, Ukraine and Uzbekistan. Croatia and Poland pointed out that they were currently in the process of developing them. From 9 countries, no information and from 7 countries only insufficient information was received by summer 2012 on the status of development of national recommendations, namely Armenia, Belarus, Bulgaria, Cyprus, Czech Republic, Greece, Israel, Kyrgyzstan, Kazakhstan, Monaco, Montenegro, Republic of Moldova, San Marino, Slovakia, Tajikistan and Turkmenistan.

**Table 1:** **National physical activity recommendations in the WHO European Region, by publication year of the most recent version and population group**

**Countries**	**Publication year**		
	**Children/young people**	**Adults**	**Older adults/elderly**
Austria [27]	2010	2010	2010
Belgium [28]	2007	2007	
Denmark [29]	n.s.	n.s.	n.s.
Estonia [30]		2006*	
Finland [31,32]	2005, 2008		
France [33]	2001*^§^	2001*^§^	
Hungary°			
Iceland [34]	2008*^§^	2008*^§^	2008*^§^
Ireland [35]	2009	2009	2009
Lithuania°			
Luxembourg [36]	n.s.	n.s.	
Malta [37]	2010	2009	
Netherlands [38]	2005*^§^	2005*^§^	2005*^§^
Norway [26]	2004	2004	
Romania°			
Russian Federation [39]		2011*^§^	
Slovenia [40]		2007	
Sweden [26]	2004	2004	
Switzerland [41]	2006*^§^	1999*^§^	
Turkey [42]		2010	
United Kingdom [43]	2011	2011	2011

n.s. = year not specified in the document, *document only available in national language, ^§^national language information could be included into the analysis, °a recommendation was reported in the country information template but could not be retrieved for analysis.

In Table 1, an overview of the national PA recommendations reported by 21 countries is shown by country and age group. Seventeen countries (81.0%) reported to have developed officially adopted national recommendations for adults of which 16 could be analyzed, while recommendations for children and youth have been developed less frequently (14 countries, 66.7%). Specific recommendations for older adults have only been developed by 6 countries (28.6%); some other countries mentioned that their recommendations for adults also referred to older age groups (e.g. Switzerland [41], Sweden/Norway [26]). It is also apparent from Table 1 that most countries except for Austria [27], the Russian Federation [39] and the United Kingdom [43] still use recommendations that were published before the release of the 2010 WHO recommendations [22].

### Children and young people

In Table 2, the analysis of the 14 recommendations for children and young people is summarized in comparison to the WHO recommendations. Thirteen countries specified duration, intensity as well as frequency in their recommendations; five were in line with the WHO recommendation on all three aspects (France [33], Ireland [35], Norway [26], Sweden [26] and the United Kingdom [43]). Compared to the WHO recommendation [22], the minimum recommended duration of activity was lower in Malta [37] and higher in Finland [31,32]. Switzerland [41] and the United Kingdom [43] also recommended more activity for young children, which are, however, not included in the WHO recommendations. The other 10 countries (as well as Switzerland for adolescents and the United Kingdom for 5–18 year olds) recommended at least 60 minutes. Two countries did not specify the recommended intensity (Finland (7–18 year olds) [31] and Iceland [34]), three countries only recommend moderate-intensity activity (Denmark [29], Luxembourg [36], Netherlands [38]), Austria recommends “at least moderate” intensity [27], and Switzerland uses a description for moderate-intensity activity through “equivalent to at least brisk walking or cycling” [41]. The WHO recommendation that some of the PA should be of vigorous intensity as well is found in Belgium [28], Denmark [29], Switzerland [41] and the United Kingdom (for 5–18 year olds) [43]; Finland recommends vigorous-intensity activity only for the youngest age group [32]. France [33] recommends vigorous-intensity activities only in relation to strength and bone health training. Three countries (Finland [31,32], Norway and Sweden [26]) also underline that PA should be varied and include movements of all major muscle groups. All countries except Belgium give specific recommendations for strength or bone health training. Five countries followed the WHO recommended frequency of at least three times per week, four countries chose to recommend such a training twice, at least twice or more vaguely “several times” a week, four countries did not specify the recommended frequency. It was also found that there is no agreement between countries with regard to minimum bouts of activity in children and young people, which was not defined by WHO for this age group: six countries (42.8%) have defined minimum bouts while Austria [27] underlines that in children and young people, any activity, no matter how short, should be counted to contribute towards the minimum recommendations. Only the recommendations of the United Kingdom for 5–18-year-olds are fully in line with the WHO recommendations.

**Table 2:** **Comparison of national physical activity recommendations for children and young people with the WHO recommendations**

**Country**	**Inactivity**	**Minimum recommendations**						**Further recommendations**
		**General recommendation**				**Additional aspects**	**Bouts**	**Add. health benefits**
		**Duration**	**Intensity**	**Frequency**	**Inclusion of vigorous intensity**			
WHO [22]	-	At least 60 minutes	Moderate- to vigorous	Daily	Should be incorporated at least 3 times/week	Activities for muscle strength and bone health at least 3 times/week	-	Amounts greater than 60 minutes daily will provide additional health benefits
Austria [27]	If sitting more than 60 minutes, activity breaks recommended	60 minutes	At least moderate	Daily	-	Activities for muscle strength and bone health at least 3 times/week, and additional activities to improve coordination and flexibility	No activity too short not to count	-
Belgium [28]	-	60 minutes	Moderate	Daily	More intense activity twice a week	-	-	-
Denmark [29]	-	At least 60 minutes	Moderate	Daily	Vigorous intensity activities of 20–30 minutes at least twice/week for fitness	Vigorous intensity activities of 20–30 minutes at least twice/week for fitness, strength, mobility and bone health	Can be divided into shorter periods	-
Finland								
<7 year olds [32]	-	At least 2 hours	Vigorous	Daily	Only vigorous intensity activities recommended	Train on a daily basis fundamental motor skills in various settings and in a diversified way. Create an environment that encourages children to be active, remove obstacles to physical activity and teach how to move safely in different environments.	-	-
7-18 year olds [31]	Avoid sitting for more than 2 hours at a time. Not more than 2 hours/day in front of entertainment media.	1-2 hours	All-round exercise	Daily	-	Exercise for bones, mobility and muscular strength at least 3 times/week	At least 10 minutes	For optimal benefit even more exercise than this should be practiced.
France [33]	-	60 minutes	Moderate to vigorous	Daily	-	At least 3 times/week vigorous activities of at least 20 minutes duration of resistance muscle training	-	-
Iceland [34]	Avoid inactivity and incorporate PA in daily life. Limit daily non-work screen time to two hours.	60 minutes	-	Daily	-	Diverse activity that promotes fitness including strength	For example 10–15 minutes	-
Ireland [35]	-	60 minutes	Moderate to vigorous	Daily	-	3 times/week strength training	-	-
Luxembourg [36]	-	60 minutes	Moderate	Daily	-	2-3 times/week strength, flexibility and balance training	-	-
Malta [37]	Decrease non-active time spent on TV, game consoles and surfing the Internet	30 to 60 minutes	Moderate or vigorous	Daily	-	For best results, include aerobic training, resistance and flexibility exercises	-	-
Netherlands [38]	-	60 minutes	Moderate	Daily	-	At least twice a week activity for physical fitness (strength, agility and coordination)	-	-
Norway [26]	-	60 minutes	Moderate to vigorous	Daily	-	Activities should be as diverse as possible to develop all aspects of physical fitness (cardio-respiratory, muscle strength, flexibility, speed, mobility, reaction time and coordination)	Can probably be divided into shorter intervals	-
Sweden [26]	-	60 minutes	Moderate to vigorous	Daily	-	Activities should be diverse, i.e. include activities on cardio-respiratory fitness, muscle strength, flexibility, speed, mobility, reaction time and coordination	Can probably be broken up into shorter bouts	-
Switzerland [41]	If sitting more than 120 minutes, activity breaks recommended	Adolescents 1 hour, younger children even longer	Equivalent to at least brisk walking or cycling	Daily	Several times/week activities for cardiovascular health	At least 10 minutes several times/week activities for muscle strength, bone health, cardiovascular health, flexibility and agility	At least 10 minutes	-
United Kingdom [43]								
0-5 years (pre-walking)	Minimize time spent being sedentary/restrained for extended periods (except sleeping)	Activity should be encouraged from birth, particularly through floor-based play and water-based activities in safe environments				-	-	-
0-5 years (walking)	Minimize time spent being sedentary/restrained for extended periods (except sleeping)	180 minutes	Moderate to vigorous	Daily	-	Movements of all the major muscle groups	-	-
5-18 years	Minimize time spent being sedentary/restrained for extended periods	At least 60 minutes and up to several hours	Moderate to vigorous	Daily	At least 3 times/week	On at least 3 days/week vigorous intensity activities including those for muscle strength and bone health	-	-

-Aspect not mentioned in the recommendation.

Six countries (namely Austria [27], Iceland [34], Finland (7–18 year olds) [31], Malta [37], Switzerland [41] and the United Kingdom [43]) also address the aspect of avoiding extended periods of inactivity or sitting. No country addressed the topic of overweight in its national recommendations for children and young people, a topic that has received much attention over recent years.

Two countries have issued separate recommendations for specific age ranges, including pre-school children (see Table 2). The WHO recommendations start at the age of 5 years.

### Adults

In Table 3, the national PA recommendations for adults are shown. In this population group, most countries follow the 1995 CDC/ACSM recommendations of “at least 30 minutes on 5 days a week” [19]; while four countries, namely Austria [27], Ireland [35], the Russian Federation [39] and the United Kingdom [43], already are consistent with the WHO recommendations of “at least 150 minutes of moderate intensity or 75 minutes of vigorous intensity PA per week, or an equivalent combination” [22]. Belgium [28] also includes the possibility to combine moderate and vigorous intensity activity to reach the minimum recommendations, but otherwise uses the 1995 recommendation of at least 30 minutes on 5 days a week, and recommends only 60 minutes of vigorous-intensity activity per week. In the Netherlands, the national “Combinorm” recommendation [38] includes a minimum recommendation of either moderate or vigorous intensity but not the possibility to combine them. Denmark requires both moderate-intensity and vigorous-intensity PA to reach the minimum recommendations [29]. All countries except France [33], the Netherlands [38] and Turkey [42] specify that the activity can be done in shorter bouts; most often at least 10 minutes are recommended in line with the WHO recommendation [22]. Four countries also quantify how much PA is recommended for additional health benefits (Austria [27], Ireland [35], the Russian Federation [39] and Switzerland [41]), whereas six countries state in general that “more activity can have additional health benefits” (Belgium [28], Denmark [29], Iceland [34], Norway [26], Sweden [26] and Switzerland [41]). Strength training is recommended by half of the countries at least two times a week, in line with the WHO recommendations. Three recommendations are fully in line with the WHO recommendations (Austria [27], the Russian Federation [39] and the United Kingdom [43]). Four countries give specific recommendations on reducing weight, avoiding weight gain or continuing weight maintenance, including Denmark [29], Norway and Sweden [26]; the most detailed recommendations on this aspect are given by Ireland [35] (see Table 3).

**Table 3:** **Comparison of national physical activity recommendations for adults with the 1995 CDC/ACSM and WHO recommendations**

**Country**	**Inactivity**	**Minimum recommendations**					**Further recommendations**		
		**Duration**		**Combination**	**Frequency**	**Bouts**	**Add. health benefits**	**Strength, balance etc.**	**Overweight**
		**Moderate intensity**	**Vigorous intensity**						
CDC/ACSM [19]	-	30 minutes or more	-	-	On most, preferably all, days of the week	Short bouts	-	-	-
WHO [22]	-	150 minutes/week	75 minutes/week	Explicitly stated	Throughout the week	At least 10 minutes	300 minutes moderate intensity/week, 150 minutes vigorous-intensity/week, or combination	Muscle-strengthening activities on 2 or more days/week	-
Austria [27]	-	150 minutes/week*	75 minutes/week (1 ¼ hours)	Explicitly stated	On most days	At least 10 minutes	300 minutes moderate intensity/week, 150 minutes vigorous-intensity/week, or combination	Muscle-strengthening activities on 2 or more days/week	-
Belgium [28]	-	30 minutes on 5 days/week (or 60 minutes if of low intensity or less than 5 days/week)	20 minutes on 3 days/week	Explicitly stated	5 or 3 days/week	At least 10 minutes	More activity can have additional health benefits	Activities for muscular strength and endurance on at least 2 days/week	-
Denmark [29]	-	At least 30 minutes ideally on 7 days/week	20-30 minutes on 2 days/week	*	Ideally on 7 days/week and 2 days/week, respectively	For example 2 × 15 minutes or 3 × 10 minutes	More activity will have additional health benefits	At least twice a week for at least 20 minutes activities of vigorous intensity for fitness, muscle and bone strength and flexibility	Moderate-intensity physical activity for at least 30 minutes, ideally 7 days/week
France [33]	-	Activity corresponding to 30 minutes of brisk walking/day	Can be carried out on an individual basis, depending on preference, capacity, health status	*-*	On most, if possible all days of the week	*-*	*-*	*-*	*-*
Iceland [34]	Avoid inactivity and incorporate physical activity into daily life.	30 minutes	In addition to basic recommendations, do at least two times a week 20-30 minutes of vigorous-intensity physical activity to promote further fitness including endurance	-	Daily	For example 10-15 minutes	More activity can promote further health benefits	At least twice per week, 20-30 minutes vigorous PA to promote further fitness including strength.	-
Ireland [35]	-	30 minutes (or 150 minutes/week) Any amount of physical activity gains some health benefits.	75 minutes/week	Explicitly stated	5 days/week	At least 10 minutes	60 minutes on 5 days/week of moderate intensity	-	To avoid weight gain: at least 350 cal./day (ca. 60 minutes brisk walking or 30 minutes jogging/day) To keep the weight off: 60-90 minutes moderate activity/day To lose weight: 1/3 more than minimum recommendation
Luxembourg [36]	-	30 minutes	-	-	Daily	Shorter bouts (e.g. 3 × 10 minutes) possible	-	2 to 3 times/week strength, flexibility and balance training	-
Malta [37]	-	30 minutes or more	-	-	On most, preferably all days of the week	Ideally at least 10 minutes but even shorter bouts contribute significantly to health benefits	-	-	-
Netherlands [38]	-	30 minutes at least 5 days/week^+^	20 minutes 3 times/week^+^	-	At least 5 days/week/3 times/week (VPA)	-	-	-	-
Norway [26]	-	30 minutes or energy expenditure of about 630 kJ	30 minutes or energy expenditure of about 630 kJ	Explicitly stated	Daily	Can probably be divided into shorter intervals, e.g. about 10 minutes	Increasing activity beyond this duration and intensity will yield additional benefits	-	More activity (ca. 60 minutes/day) of moderate and/or vigorous intensity may be needed for prevention of weight gain
Russian Federation [39]	Avoid sedentary behaviour	150 minutes/week (2 ½ hours)	75 minutes/week (1 ¼ hours)	Explicitly stated	Preferably spread across the week	At least 10 minutes	300 minutes (5 hours) moderate intensity/week, 150 minutes (2 ½ hours) vigorous-intensity/week, or combination	At least 2x/week strength training	-
Slovenia [40]	-	30 minutes	-	-	At least 5 days/week	Not shorter than 10-15 minutes	-	Exercise should be divided: 50% aerobic, 25% flexibility, 25% muscular strength	-
Sweden [26]	-	30 minutes or energy expenditure of about 630 kJ	30 minutes or energy expenditure of about 630 kJ	Explicitly stated	Daily	Can probably be divided into shorter intervals, e.g. about 10 minutes	Increasing activity beyond this duration and intensity will yield additional benefits	-	More activity (ca. 60 minutes/day) of moderate and/or vigorous intensity may be needed for prevention of weight gain
Switzerland [41]	-	30 minutes	-	-	Daily or most days of the week	At least 10 minutes	Additional effects achieved with more activity. Already active individuals can achieve additional health benefits with targeted training (at least 3 d/wk. 20-60 minutes with high intensity)	Strength training (8-15 repetitions) and flexibility training, gymnastics and stretching exercises twice a week	-
Turkey [42]	-	30 minutes	-	-	Daily	-	-	-	Be physically active every day, do these activities slowly
United Kingdom [43]	Minimize sedentary (sitting) time for extended periods	150 minutes; one way to approach this is to do 30 minutes on at least 5 days/week	75 minutes	Explicitly stated	Daily (MPA) Spread across the week (VPA)	At least 10 minutes	-	Strength training at least 2 days/week	-

-Aspect not mentioned in the recommendations.

^+^Adherence to either the moderate- or vigorous intensity recommendation, not a combination. *Both the moderate- and vigorous intensity recommendations required to meet recommendations.

MPA = moderate physical activity; VPA = vigorous physical activity.

### Older adults

In Table 4, the national PA recommendations for older adults from six countries are compared with the 2010 WHO recommendations [22]. The nationally recommended amount of activity is similar to that for adults with regard to duration, frequency and intensity in all six countries and thus corresponds to the WHO recommendations [22]. WHO and all countries but Denmark [29] and Iceland [34] underline that for this age group some activity is better than none and that also less than the minimum recommended amount of activity has important health benefits. All countries but the Netherlands [38] also make recommendations for strength training and all but Iceland [34] and the Netherlands [38] recommend balance training. On the latter, the United Kingdom [43] as well as WHO [22] state that such training is specifically recommended for subjects at risk of falls.

**Table 4:** **Comparison of national physical activity recommendations for older adults with the WHO recommendations**

**Country**	**Inactivity**	**Minimum recommendations**					**Further recommendations**		
		**Duration**		**Combination**	**Frequency**	**Bouts**	**Add. health benefits**	**Strength, balance etc.**	**Overweight**
		**Moderate intensity**	**Vigorous intensity**						
WHO [22]	-	150 minutes/week, or be as physically active as abilities and conditions allow	75 minutes/week	Explicitly stated	Throughout the week	At least 10 minutes	300 minutes moderate intensity/week, 150 minutes vigorous-intensity/week, or combination	- Persons with poor mobility: activity to enhance balance and prevent falls on 3 or more days/week; - muscle-strengthening activities on 2 or more days/week	-
Austria [27]	-	150 minutes/week Be as active as condition allows if minimum recommendations cannot be met	75 minutes/week	Explicitly stated	On most days of the week	At least 10 minutes	300 minutes moderate intensity/week, 150 minutes vigorous-intensity/week, or combination	On 2 or more days/week: strength training, balance training	-
Denmark [29]	-	At least 30 minutes	-	-	Daily	-	More activity will have additional health benefits	- Activities to maintain and enhance muscle strength and fitness at least twice/week for 20 minutes - Balance and stretching twice a week at least 10 minutes	-
Iceland [34]	Avoid inactivity and incorporate physical activity into daily life	30 minutes	-	-	Daily	For ex. 10-15 minutes	More activity can promote further health benefits. In addition to basic recommendations, at least twice a week 20-30 minutes of vigorous activities to promote further fitness.	Strength training is especially beneficial for older people	-
Ireland [35]	-	30 minutes daily or 150 minutes/week Any amount of physical activity gains some health benefits	-	-	5 days/week	At least 10 minutes	-	Strength and balance training on 2-3 days/week	-
Netherlands [38]	-	30 minutes at least 5 days/week^+^ For non-active people, all extra physical exercise is significant, regardless of intensity, duration, frequency and type	20 minutes 3 times/week	-	Daily	-	-	-	-
United Kingdom [43]	Minimize sedentary (sitting) time for extended periods	Any amount of activity gains some health benefits. Some activity is better than none, and more activity provides greater benefits. 150 minutes (2½ hours); one way to approach this is 30 minutes on at least 5 days/week	75 minutes	Explicitly stated	Daily (MPA)Spread across the week (VPA)	At least 10 minutes	-	Strength training on at least 2 days/week Older adults at risk of falls: balance and coordination training on at least 2 days/week	-

^+^Adherence to either the moderate- or vigorous intensity recommendation, not a combination.

-Aspect not mentioned in the recommendations. MPA = moderate physical activity; VPA = vigorous physical activity.

## Discussion

This paper presents the first overview and systematic analysis of information on national PA recommendations for the WHO European Region. It includes also information from five countries where information was not available in English, which is often omitted in international analyses. This allows drawing some general conclusions even though it has to be borne in mind that limited information was available from about one third of the 53 countries in the WHO European Region, mostly from the central and eastern part. It is also possible that despite the thorough search process, some national recommendations could not be identified, and for some countries, the situation may have changed since the information was collected.

It is noteworthy to mention that it was a challenging process to obtain information on the existence for national recommendations and to gain access to the source documents. In many cases, the documents were only available in national languages and not online. In order to facilitate the collation of such overviews as this paper and thus information sharing between countries, it would be preferable to publish at least an English summary of the national recommendations and to make them easily available.

Only slightly more than half of the countries for which information was available had developed national PA recommendations by 2011 and few pointed out that they were in the process of developing them. It is likely that some of the non-responding countries also had not developed national recommendations yet and thus probably less than 40% of all 53 countries in the European Region. Organizations such as WHO or networks such as HEPA Europe [44] can play an important role in facilitating information sharing between countries and in supporting the process to develop a national recommendation. Furthermore, it is noticeable that more countries from northern and western Europe reported on their national recommendations, pointing to an uneven distribution regarding the state of development of PA promotion across the Region. These results show that there are further investments needed to address this important step which is often the first one towards a comprehensive approach to promoting PA by establishing a nationally agreed benchmark for stakeholders and the population to refer to, either by developing national recommendations or by endorsing already established international ones. Agreeing such a benchmark can also support the development and implementation of related monitoring and surveillance to evaluate the achievement of the nationally agreed levels of physical activity.

Not surprisingly, the majority of the recommendations for adults still follow the 1995 CDC/ACSM recommendations. While in many aspects they are still consistent with the updated American or global recommendations, important differences exist. Thus, there is a need for these countries to consider updating their recommendations in light of the most recent evidence on PA and health on which the current WHO recommendations are based [22], in particular with regard to weekly rather than daily recommended durations of activity, a stronger emphasis on vigorous-intensity PA and the possibility to combine moderate- and vigorous-intensity PA to reach the minimum recommendations. When doing so countries may also consider on how to best bring the updated message to the public to avoid weakening the PA promotion message. For example, often used slogans such as “5 times 30” may be kept as one example of how the new recommendations can be reached, amongst others.

Only two thirds of the 21 countries that have developed national PA recommendations targeted children and young people. While these recommendations were mostly in line with the current WHO recommendations [22], disagreements were found in particular with regard to recommended intensities and minimum bouts of activity. In view of the high prevalence of insufficient PA levels in this population group [3], specific recommendations for this age group can be an important instrument for policy makers and practitioners to support investments in this important issue. A few countries also addressed pre-school age groups or toddlers [32,43]. While evidence on health effects is scarcer for these ages, these as well as other examples found outside the Region [45,46] can serve as first illustrations for others to build on as information starts to grow.

Even less frequently than young people, older adults have been addressed by specific recommendations. In view of ageing societies in many European countries and the strong evidence on positive effects of PA on physical, social and mental health specifically also in older people [47,48], it is of particular importance to use national recommendations for this age group as advocacy tool and as basis for implementation of specific programs.

Finally, the analysis showed that avoiding extended periods of inactivity or sitting has only been addressed by few countries. The mounting evidence of its detrimental health effects for young people [49] as well as adults [50] warrants a stronger emphasis on this issue. PA recommendations are also only rarely issued with regard to preventing or addressing overweight; it can be speculated that this aspect might be more frequently addressed as part of nutrition recommendations rather than PA recommendations.

## Conclusions

Overall, our analysis showed that while progress has been made in the development of national PA recommendations, the conclusions made by Oja et al. [23] regarding the need to review the existing PA recommendations at the European level and assess their consistency with the new evidence and the new recommendations are still valid. Much remains to be done for the new WHO recommendations to be fully reflected in national documents across all parts of the European Region and for all age groups. In addition, avoiding extended periods of inactivity and overweight are only addressed by a minority of countries yet.

## Abbreviations

ACSM: American College of Sports Medicine
CDC: Centers for Disease Control and Prevention
EC: European Commission
HEPA: Health-enhancing physical activity
NCDs: Noncommunicable diseases
PA: Physical activity
WHO: World Health Organization
